# Assessing the accuracy of Seismofit® as an estimate of preoperative maximal oxygen consumption in patients with hepato-pancreato-biliary, colorectal, and gastro-oesophageal cancer

**DOI:** 10.1016/j.bjao.2025.100395

**Published:** 2025-04-08

**Authors:** Nicholas Tetlow, Philip Devendra, James Waiting, Maria Aresu, Abena Glover, Martin Rooms, Shaman Jhanji, Don Milliken

**Affiliations:** 1 Department of Perioperative Medicine, Anaesthesia, Pain and Critical Care, The Royal Marsden NHS Foundation Trust, London, UK; 2 Centre for Peri-operative Medicine, Department of Targeted Intervention, Division of Surgery and Interventional Science, University College London, London, UK; 3 Department of Anaesthetics, Sheffield Teaching Hospitals NHS Foundation Trust, Sheffield, UK; 4 Research Data & Statistics Unit, Royal Marsden Clinical Trials Unit, The Royal Marsden NHS Foundation Trust, London, UK

**Keywords:** Accuracy, CPET, major abdominal cancer surgery, peak oxygen uptake, seismocardiography

## Abstract

**Background:**

Peak oxygen uptake (VO_2_ peak) measured during cardiopulmonary exercise testing (CPET) is commonly used to objectively assess fitness and inform risk stratification. Preoperative CPET is not always universally available. Seismofit® offers a noninvasive, non-exercise alternative for estimating VO_2_ peak, though it has not been validated in patients awaiting major abdominal cancer surgery.

**Methods:**

Prospective single-centre blinded observational study in patients with hepato-pancreato-biliary, colorectal, or gastro-oesophageal cancer undergoing preoperative assessment. Patients underwent Seismofit® assessment before routine CPET. Primary outcome was the relationship between Seismofit*®-*estimated VO_2_ peak and CPET-measured VO_2_ peak. Secondary outcomes explored the relationship between Seismofit® and CPET for (i) bias and agreement limits; (ii) surgical subgroup; (iii) commonly reported CPET variables; (iv) patient acceptance.

**Results:**

Thirty-three participants (median [interquartile range] age: 67 yr [58–75 yr]; 20 [61%] males) completed both CPET and Seismofit®. No linear association was found between Seismofit*®-*estimated VO_2_ peak and CPET-measured VO_2_ peak: Pearson *r*=0.111 (95% confidence interval −0.242 to 0.437), *R*^2^=0.012, *P*=0.539. Compared with CPET, Seismofit® demonstrated a large bias (standard deviation) 12.8 (8.8); 95% limits of agreement (−4.5 to 30.0). No association existed between Seismofit*®-*estimated VO_2_ peak and CPET-measured VO_2_ peak in the hepato-pancreato-biliary or gastro-oesophageal subgroup or between Seismofit®-estimated VO_2_ peak and commonly reported CPET variables.

**Conclusions:**

There was no evidence of linear association between Seismofit®-estimated VO_2_ peak and objectively measured VO_2_ peak by CPET in patients undergoing assessment for major abdominal cancer surgery. This finding was consistent across all subgroup and exploratory analyses. Seismofit® tended to overestimate VO_2_ peak with a high degree of bias.

**Clinical trial registration:**

NCT05831488.

Patients under consideration for major hepato-pancreato-biliary (HPB), colorectal, and gastro-oesophageal cancer surgery should undergo thorough preoperative assessment of functional capacity.^1^ Cardiopulmonary exercise testing (CPET) uses graded exercise to provide an integrated assessment of cardiorespiratory fitness, and is recommended in patients undergoing higher-risk procedures.^2^^,^^3^ Widespread application of preoperative CPET has been limited by the requirement for specialist training, need for expensive equipment, and dedicated space.^4^ CPET requires patients to perform intense exercise and, whilst considered safe, carries a risk of an event requiring hospitalisation of 2 per 1000 tests and death of 2–5 per 100 000 tests.^5^

Peak oxygen uptake (VO_2_ peak), defined as the highest oxygen uptake attained during CPET, may be considered to define cardiorespiratory fitness. Greater cardiorespiratory fitness is associated with a variety of protective factors associated with increased resilience to surgical stress.^6^ Importantly, VO_2_ peak has been shown to predict perioperative morbidity and mortality in patients undergoing major abdominal surgery.^7^ Meta-analysis of 52 studies (*n*=10 030) demonstrated an inverse association between VO_2_ peak and the frequency of perioperative complications.^8^

The Centre for Peri-Operative Care recommends routine screening for reduced functional capacity using a validated tool.^1^ The most commonly used tool is the Duke Activity Status Index (DASI),^9^^,^^10^ but concerns regarding the accuracy of the DASI exist as it is a self-reported subjective assessment. Comparison of DASI-derived VO_2_ peak with objectively measured VO_2_ peak from CPET in 623 patients undergoing preoperative assessment demonstrates that DASI systematically overestimates fitness when compared with CPET.^11^ There is a need for objective and accessible risk-stratification tools to identify this ‘high-risk’ group.

Seismofit® is a commercially available device that uses seismocardiography (SCG) to provide a non-exercise, noninvasive estimate of VO_2_ peak. Fiducial points in the resting SCG signal correlate with events in the cardiac cycle^12^ and provide a valid estimate of VO_2_ peak in non-clinical populations.^13^^,^^14^ Seismofit® offers potential advantages over CPET including lower cost and minimal training, however its use in the assessment of fitness in patients with cancer awaiting major abdominal surgery has not been validated.

This study aimed to assess the relationship between the Seismofit®-derived VO_2_ peak estimate and CPET-measured VO_2_ peak in patients undergoing routine preoperative CPET. The aim was to establish whether Seismofit® could be used as a less resource-intensive and better tolerated alternative to CPET, or whether it might be useful as a screening tool to efficiently identify patients with exercise intolerance who may benefit from further characterisation by CPET.

## Methods

### Study design

We conducted a prospective, single-centre, non-randomised observational study at The Royal Marsden Hospital NHS Foundation Trust. Ethical approval was given by Preston Research Ethics Committee (REC Reference 22/NW/0385; IRAS project ID 309212) on 13 January 2023 and conducted in accordance with the principles of the Declaration of Helsinki and the Research Governance Framework. The study was prospectively registered (NCT05831488) on 17 January 2023. Recruitment and data collection took place between 30 March 2023 and 25 July 2023. Written informed consent was obtained from all patients.

#### Study participants

We included adults aged ≥18 yr, scheduled for elective surgery for resection of HPB, colorectal or gastro-oesophageal primary or secondary cancer. Exclusion criteria were as follows: subjects unable to give voluntary written informed consent to participate; diagnosis of stenosis or regurgitation of any cardiac valve of moderate or greater severity; previous aortic or mitral valve surgery, valvuloplasty or transcatheter valve implantation; permanent pacemaker or cardiac resynchronisation device *in situ*; diagnosis of severe pulmonary hypertension; permanent atrial fibrillation.

### Conduct of the study

All recruited patients who underwent Seismofit® before CPET were considered for inclusion in the analysis. Patients were excluded from analysis if they had more than two ectopic beats during a 10 s period during rest or were found not to be in sinus rhythm on continuous 12-lead ECG monitoring during the resting and unloaded phase of CPET. Any patients who did not achieve maximal CPET according to conventional criteria^5^ and physiologist experience were excluded from the analysis.

### Measurements and data handling

#### Seismofit® estimation of VO_2_ peak

The Seismofit® system consists of a small device containing a three-axis accelerometer, a smart-phone app, and cloud-based signal processing. Before estimation, participant age, height, weight, and sex were entered into the app. Participants were asked to confirm that they had not undertaken strenuous activity in the 10 min before measurement. Otherwise, a 10 min rest period was provided. The SCG recording was performed with the Seismofit® device mounted on the sternum with double-sided adhesive tape, with participants in a supine position and at rest. VO_2_ peak was estimated using the latest Seismofit*®* estimation model (SCG Version 4.3). All Seismofit® measurements took place before CPET.

#### Cardiopulmonary exercise testing

CPET was performed in accordance with the Peri-Operative Exercise Testing and Training Society (POETTS) guidelines.^5^ Exercise testing was carried out on an electromagnetically braked cycle-ergometer (Ergoselect 200, Ergoline GmbH, Bitz, Germany) and breath-by breath gas-exchange analysis was performed by a metabolic cart (Ultima CardiO_2_®; Medical Graphics Corp., St Paul, MN, USA) using Breeze Suite (8.6.0.93 SP6) which was calibrated before each use. Continuous 12-lead ECG was measured using Mortata Xscribe (v4.0.4). Testing consisted of rest (3 min), unloaded cycling (3 min), ramped exercise (∼8–12 min), and recovery (3 min). The ramp protocol was selected by the physiologist based on experience, patient weight, age, sex, activity levels, and haemoglobin concentration.

Oxygen uptake (VO_2_) and carbon dioxide production (VCO_2_), work-rate, ventilatory frequency, tidal volume, and end-tidal gas tensions were recorded. Ventilatory equivalents for oxygen (V_E_/VO_2_) and carbon dioxide (V_E_/VCO_2_) were derived. Oxygen uptake at anaerobic threshold (AT) was determined using a combination of the V-slope method, changes in ventilatory equivalent and end-tidal profiles as per POETTS guidance.^5^ Values for V_E_/VCO_2_ were recorded at AT or their nadir if AT could not be determined. VO_2_ peak was defined as the highest VO_2_ over the last 10 s of ramped exercise. Peak power output (PPO) was calculated by dividing peak work (Watts) by patient weight (kg). VO_2_ peak and AT were adjusted for body mass (ml kg^−1^ min^−1^). All CPET variables recorded were determined by a POETTS accredited physiologist and independently verified by a POETTS accredited clinician, with any disagreement adjudicated by a third practitioner, a POETTS accredited clinician, whose decision was final.

#### Blinding

To avoid bias in the interpretation of the data, the physiologist carrying out CPET was blinded to the Seismofit® prediction. Seismofit® recording was carried out by another trained staff member where available and not reviewed until after CPET completion.

#### Patient acceptance questionnaire

For each assessment, patients reported their degree of concern (no concern, little, moderate, intense, very intense) and comfort and overall satisfaction (very good, good, barely acceptable, poor, very poor). Patients recorded pain during the assessment using a visual analogue scale (0–100). Willingness to undergo the test again (yes, no, don't know) and which test the participant would prefer (Seismofit®, CPET, undecided) were recorded.

### Outcome measures

#### Primary outcome

The primary outcome was the linear association between Seismofit®-estimated VO_2_ peak and CPET-measured VO_2_ peak.

#### Secondary outcomes

1. Limits of agreement and average bias between Seismofit®-estimated VO_2_ peak and CPET-measured VO_2_ peak.
2. Linear association in each surgical subgroup: HPB, gastro-oesophageal, and colorectal cancer patients.
3. Linear association between Seismofit®-estimated VO_2_ peak and the CPET-measured variables: positive association with AT and PPO and negative association with V_E_/VCO_2_.
4. Patient concern, comfort, pain score, and overall satisfaction of Seismofit® measurement compared with CPET.

### Exploratory analyses

We assessed the linear association between Seismofit®-estimated VO_2_ peak and CPET-measured VO_2_ peak for patients without clinically significant anaemia ([Hb] >100 g L^−1^). We also assessed linear association in the cohort excluding patients with low (<20 kg m^−2^) and high (>35 kg m^−2^) BMI.

### Sample size calculation

The observed correlation between the Seismofit® device and CPET in non-clinical participants is 0.9.^13^ Using a two-sided Fisher's z test, with null hypothesis ρ=0.7 a sample size of 32 participants was required to detect a ρ=0.9 at the 5% significance level with 90% power (nQuery version 8.7).

### Statistical analyses

Statistical analysis and graph preparation was performed using GraphPad Prism (Version 10.3.0, GraphPad Software, San Diego, CA, USA). Normality was assessed using the Shapiro–Wilk test. Data were presented as mean (standard deviation [sd]) or median (interquartile range [IQR]) for normally and non-normally distributed continuous data, respectively. Categorical data were presented as number (%). Differences between groups were assessed using paired *t*-tests or Wilcoxon matched-pairs signed rank tests as appropriate. Linear associations were assessed using the Pearson correlation coefficient or, for non-parametric data, the Spearman correlation. Limits of agreement (95% limits) and bias (sd) between Seismofit®-estimated VO_2_ peak and CPET-measured VO_2_ peak were assessed using Bland–Altman analysis. For the subgroup analysis by surgical cohort, non-parametrical tests were used and a pre-specified minimum of 10 participants was required per cohort. Two-sided tests were used throughout with significance set at *P*≤0.05. All confidence intervals (CI) are 95% and two-sided.

## Results

### Study characteristics

**Figure 1:**
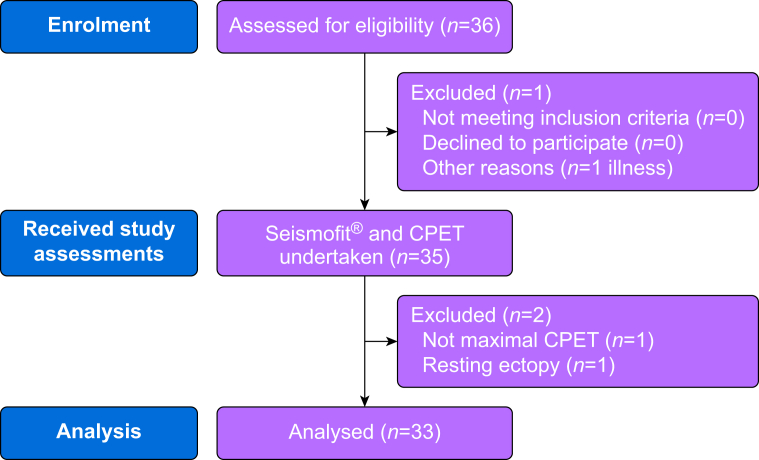
Study flow diagram displaying number of patients enrolled, receiving assessments and analysed. CPET, cardiopulmonary exercise testing.

Thirty-six patients met the inclusion criteria and gave consent to participate. One patient was unable to perform CPET after Seismofit® assessment because of illness. Thirty-five patients underwent Seismofit® assessment and CPET between 30 March 2023 and 25 July 2023. Two patients were withdrawn from the analysis: one had more than two ectopic beats during a 10 s period on resting ECG and one patient did not achieve a maximal CPET (Fig 1). Patient characteristics are presented in Table 1.

**Table 1:** Baseline characteristics of patients included in he analysis who underwent Seismofit® assessment and CPET. Values are presented as median (interquartile range), mean (standard deviation) or number (%). ASA-PS, American Society of Anesthesiologists physical status; AT, anaerobic threshold; BMI, body mass index; COPD, chronic obstructive pulmonary disease; CPET, cardiopulmonary exercise testing; PPO, peak power output; V_E_/VCO_2_, ventilatory equivalent for carbon dioxide. ∗AT was unable to be determined in one patient (*n*=32).

Variable	*n*=33
Characteristics	
Age, yr (range)	67 (58–75)
Sex	
Male	20 (61)
BMI (kg m^−2^)	26.3 (22.8–30.7)
Haemoglobin (g L^−1^)	124 (119–135)
ASA-PS grade	
1	1 (3)
2	15 (46)
3	17 (52)
Planned surgery	
Hepato-pancreato-biliary	13 (39)
Gastro-oesophageal	12 (36)
Colorectal	8 (24)
Operation	
Major colonic or rectal resection	6 (18)
Total pelvic exenteration	1 (3)
Small bowel resection	2 (6)
Liver resection	4 (12)
Whipple's procedure	5 (15)
Distal pancreatectomy and spleen	1 (3)
Total/partial gastrectomy	7 (21)
Oesophagogastrectomy	4 (12)
Other	3 (9)
Neoadjuvant therapy	
Chemotherapy	23 (70)
Radiotherapy	8 (24)
Medical history	
Hypertension	11 (33)
COPD	4 (12)
Asthma	1 (3)
Myocardial infarction	1 (3)
Cerebrovascular accident	1 (3)
Diabetes mellitus	9 (27)
Smoking status	
Current	4 (12)
Former	13 (39)
Never	16 (49)
Cardiorespiratory fitness	
VO_2_ peak (ml kg^−1^ min^−1^)	17.7 (3.2)
AT (ml kg^−1^ min^−1^)	9.9 (1.7)∗
V_E_/VCO_2_	32.0 (29.5–33.5)
PPO (W kg^−1^)	1.4 (0.3)

### Primary outcome

Linear association between Seismofit®-estimated VO_2_ peak and cardiopulmonary exercise testing-measured VO_2_ peak.

**Figure 2:**
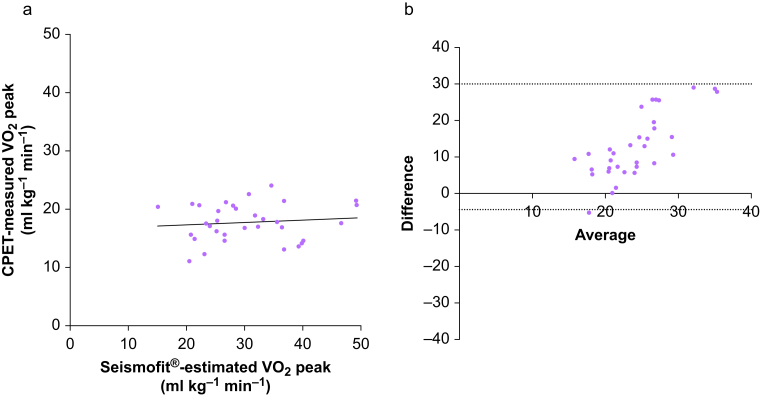
Comparison of Seismofit® device and CPET for VO_2_ peak for (a) linear association between Seismofit®-estimated VO_2_ peak and CPET-measured VO_2_ peak (ml kg^−1^ min^−1^) and (b) Bland–Altman analysis of 33 paired measurements demonstrating difference (Seismofit®-estimated VO_2_ peak–CPET-measured VO_2_ peak) *vs* average with dotted lines indicating 95% limits of agreement. CPET, cardiopulmonary exercise testing; VO_2_ peak, peak oxygen uptake.

There was no linear association between Seismofit*®-*estimated VO_2_ peak and CPET-measured VO_2_ peak (Fig 2a): Pearson *r* 0.111 (95% CI −0.242 to 0.437), *R*^2^=0.012, *P*=0.539. Mean (sd) (ml kg^−1^ min^−1^) for Seismofit®-estimated VO_2_ peak was 30.5 (8.6) *vs* 17.7 (3.2) for CPET-measured VO_2_ peak; *P*<0.0001.

### Secondary outcomes

#### Limits of agreement and average bias between Seismofit®-estimated VO_2_ peak and cardiopulmonary exercise testing-measured VO_2_ peak

Bland–Altman analysis demonstrated poor agreement (Fig 2b) between Seismofit®-estimated VO_2_ peak and CPET-measured VO_2_ peak. Comparison of the difference (A–-B) *vs* average for A: Seismofit®-estimated VO_2_ peak and B: CPET-measured VO_2_ peak, demonstrated a large bias (sd) 12.8 (8.8) 95% limits of agreement (−4.5 to 30.0).

#### Strength and direction of relationship between Seismofit®-estimated VO_2_ peak and CPET-measured VO_2_ peak in each surgical subgroup: HPB, gastro-oesophageal, and colorectal

##### Hepato-pancreato-biliary cohort

**Figure 3:**
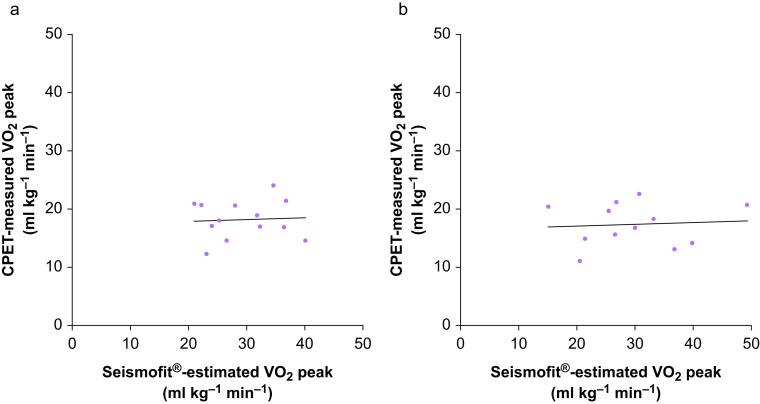
Strength and direction of relationship between Seismofit®-estimated VO_2_ peak and CPET-measured VO_2_ peak in (a) HPB subgroup; *n*=13 and (b) gastro-oesophageal subgroup; *n*=12. CPET, cardiopulmonary exercise testing; HPB, hepato-pancreato-biliary; VO_2_ peak, peak oxygen uptake.

Spearman correlation demonstrated no evidence of association between Seismofit*®-*estimated VO_2_ peak and CPET-measured VO_2_ peak for the HPB subgroup (Fig 3a): Spearman *r*=−0.036 (95% CI −0.588 to 0.539); *P*=0.910. Median (IQR) (ml kg^−1^ min^−1^) for Seismofit®-estimated VO_2_ peak was 28.0 (23.6–35.5) *vs* 18.0 (15.8–20.8) for CPET-measured VO_2_ peak; *P*=0.0002.

##### Gastro-oesophageal cohort

Spearman correlation demonstrated no evidence of association between Seismofit*®-*estimated VO_2_ peak and CPET-measured VO_2_ peak (Fig 3b): Spearman *r*=−0.091 (95% CI −0.524 to 0.643) *P*=0.783. Median (IQR) (ml kg^−1^ min^−1^) for Seismofit®-estimated VO_2_ peak was 28.4 (22.4–35.9) *vs* 17.6 (14.3–20.6) for CPET-measured VO_2_ peak; *P*=0.0010.

##### Colorectal cohort

We did not assess the relationship between Seismofit®-estimated VO_2_ peak and CPET-measured VO_2_ peak in the colorectal subgroup because the sample size was less than our pre-specified minimum.

#### Strength and direction of relationship between Seismofit®-estimated VO_2_ peak and the cardiopulmonary exercise testing-measured variables of AT, V_E_/VCO_2_, and PPO

Of the 33 participants who completed Seismofit® and CPET, we were unable to determine the AT in one participant (3%). There was no evidence of linear association between Seismofit*®-*estimated VO_2_ peak and CPET-measured variables of (i) AT (*n*=32): Pearson *r*=0.167 (95% CI −0.193 to 0.487), *R*^2^=0.028, *P*=0.362; (ii) V_E_/VCO_2_: Spearman *r*= 0.110 (95% CI −0.252 to 0.446), *P*=0.541, and (iii) PPO: Pearson *r*=0.162 (95% CI −0.192 to 0.478), *R*^2^=0.026, *P*=0.368.

#### Patient concern, comfort, overall satisfaction, and pain score of Seismofit® measurement compared with cardiopulmonary exercise testing

**Figure 4:**
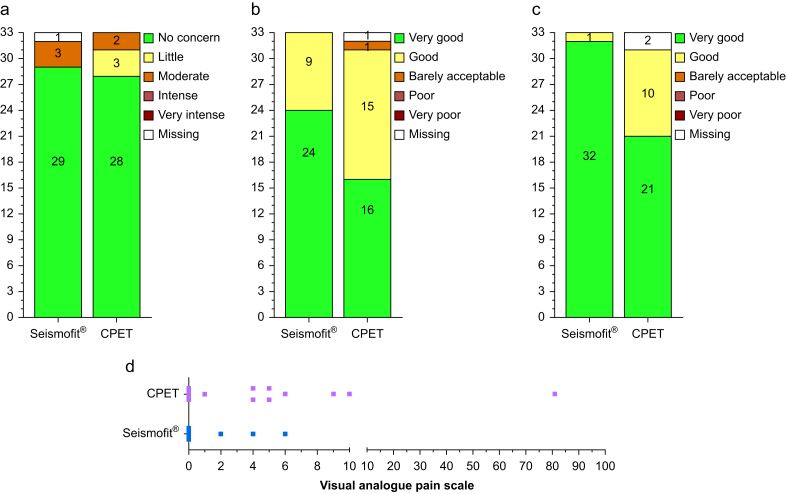
Participant questionnaire responses for Seismofit® and CPET demonstrating (a) concern about the test; (b) comfort during the test; (c) overall satisfaction, and (d) pain during the test. CPET, cardiopulmonary exercise testing.

All 33 patients received the patient acceptance questionnaire and the completion rate was high for both Seismofit® (97%) and CPET (91%). Most patients reported ‘no concern’ for both Seismofit® (*n*=29/32, 91%) and CPET (*n*=28/33, 85%) (Fig 4a). A higher number of patients responded ‘very good’ regarding comfort for Seismofit® (*n*=24/33, 73%) *vs* CPET (*n*=16/32, 50%), with one patient (*n*=1/32, 3%) reporting ‘barely acceptable’ for CPET (Fig 4b). For overall satisfaction, more patients responded ‘very good’ for Seismofit® (*n*=32/33, 97%) compared with CPET (*n*=21/31, 67%) (Fig 4c). The median (range) pain score was 0 (0–6) for Seismofit® and 0 (0–81) for CPET (Fig 4d), with only one patient reporting a pain score of >10 which was for CPET.

For ‘willingness to undergo the test again’, all patients were willing to undergo Seismofit® again (*n*=33/33, 100%) and all patients were willing to undergo CPET again (*n*=32/32, 100%). Future test preference was similar between Seismofit® (42%) and CPET (42%) with 15% remaining undecided.

### Exploratory analysis

Among patients without clinically significant anaemia on the day of assessment (haemoglobin >100 g L^−1^), there was no linear association between Seismofit*®-*estimated VO_2_ peak and CPET-measured VO_2_ peak (*n*=30): Pearson *r*= 0.106 (95% CI −0.264 to 0.449), *R*^2^=0.011, *P*=0.577. Excluding patients with low (<20 kg m^−2^) and high (>35 kg m^−2^) BMI, there was no linear association between Seismofit*®-*estimated VO_2_ peak and CPET-measured VO_2_ peak (*n*=26): Pearson *r*=0.186 (95% CI −0.217 to 0.535), *R*^2^=0.035, *P*=0.363.

## Discussion

We demonstrated no association between VO_2_ peak estimated by the Seismofit® device and VO_2_ peak objectively measured by CPET in patients undergoing assessment for major abdominal cancer surgery. This finding was consistent across all subgroup analyses. Compared with CPET, Seismofit® tended to overestimate VO_2_ peak with a high degree of bias.

These findings are unexpected and inconsistent with those of Sorensen and colleagues^13^ who demonstrated a strong positive correlation of 0.90 (95% CI 0.83–0.94) also using an algorithm including SCG features, sex, age, height, and mass. Our findings also contrast with the recent study of Hansen and colleagues,^14^ who observed a correlation of 0.79 between Seismofit®-predicted and CPET-measured VO_2_max. Importantly, the Seismofit® algorithm's development cohort and the subjects in both of these previous validation studies were drawn from healthy, non-clinical populations. Patients in our study demonstrated much poorer exercise performance than subjects in those, with a mean CPET-measured VO_2_ peak of 17.7 ml kg^−1^ min^−1^, compared with ∼32 ml kg^−1^ min^−1^ in Sorensen and colleagues^13^ and ∼39 ml kg^−1^ min^−1^ in Hansen and colleagues.^14^ Performance of multivariable prediction models such as those underlying the Seismofit® estimates tends to be poorer when predicting values in subjects that differ substantially from their development cohort.^15^ However, the average CPET parameters reported in our study for AT, V_E_/VCO_2_, VO_2_ peak, and PPO (Table 1) are comparable to those reported for patients undergoing preoperative CPET^16^ and representative of the population intended for device validation.

One of the most influential covariates that Seismofit® uses to predict VO_2_max is the FRIEND equation-predicted VO_2_max.^17^ The FRIEND model was developed using a large cohort of healthy volunteers, and their final model includes age, sex, height, and weight. This model has demonstrated good performance on internal validation but has not yet been externally validated. The FRIEND study has also published the distribution of VO_2_ max values for their cohort, stratified by age and sex.^18^ Comparison with our study reveals that 13 of the 33 (39%) patients in our cohort achieved a VO_2_max that was below the 5th percentile of age- and sex-matched FRIEND study subjects. We would expect that the FRIEND prediction model would perform poorly in predicting the extreme values observed in our cohort, and that, as an important covariate in the Seismofit® model, it would adversely affect the Seismofit® estimate.

Poor performance of Seismofit® in our study could be further explained by differences in the factors limiting exercise performance in our patients and of those used to develop and validate Seismofit®. During maximal exercise in normal, healthy subjects, there is excess capacity of skeletal muscle mitochondria to utilise oxygen delivered by the cardiovascular system.^19^ Oxygen delivery, dependent primarily on cardiac performance, is the rate-limiting constraint, and this provides the theoretical basis for the Seismofit® device's use of SCG. However, emerging evidence demonstrates that exercise capacity in untrained or deconditioned individuals, such as the patients in this study, is primarily limited by capacity of the mitochondria to consume oxygen, despite an excess of oxygen supply.^20^

Exercise intolerance in this cohort may also be influenced by cancer metabolism^21^ and skeletal muscle wasting, which is common in cancer cachexia.^22^ Neoadjuvant therapies also impair cardiorespiratory fitness^23^^,^^24^ by altering mitochondrial oxidative phosphorylation^25^ and may confound the relationship between resting cardiac performance and maximal exercise performance, undermining the basis for the Seismofit® device's predictions. This remains an untested hypothesis which would require further study. Equally, differences in Seismofit® performance between our cohort and the participants in the Seismofit® validation studies may be largely because our patients were older and more deconditioned.

Strengths of the study include the use of blinding and independent verification of all CPET variables. Our study is limited by the use of a cycle-ergometer over treadmill. The FRIEND model's development cohort consisted entirely of subjects who underwent treadmill-based CPET. Maximum oxygen uptake is reported to be 5–20% lower on a cycle-ergometer than on the treadmill.^26^^,^^27^ The FRIEND model and, therefore, the Seismofit® model, may overestimate VO_2_max in cycle ergometer-tested patients such as those in our study. This may help to explain the positive bias encountered in our study. However, the majority (93%) of UK centres using CPET variables to guide perioperative counselling use cycle ergometer-based testing.^4^ Although CPET-measured VO_2_ peak can be compared with an estimated VO_2_ peak from Seismofit®, VO_2_ peak alone is not a ‘gold standard’ measure of functional capacity. Although this comparative approach enables appropriate statistical comparison, it overlooks how CPET data, including VO_2_ peak, are used in everyday clinical practice. Emphasis is often also placed on other prognostic cardiopulmonary variables, including their dynamic responses. These combined factors guide risk stratification, perioperative management, and prehabilitation planning beyond VO_2_ peak alone.

Preoperative evaluation presents a clear case for a relatively inexpensive, scalable device such as Seismofit® and our study confirms the device's acceptability with patients reporting high comfort and overall satisfaction. One patient experienced significant arthritic knee pain during CPET, finding it barely acceptable. In patients with mobility issues, a non-exercise alternative such as Seismofit® would be pragmatic. Our study does not support the use of Seismofit® in the estimation of VO_2_ peak for patients with cancer awaiting major abdominal surgery. Any future use in this context requires redevelopment of the multivariable model underlying the device's predictions and subsequent robust validation in relevant patient groups.

## Authors’ contributions

Conception: NT, PD, DM

Study design: NT, PD, DM

Data acquisition: NT, JW

Analysis: NT, MA, AG, DM

Drafting article: NT, JW, DM with input from all authors

Manuscript revision: all authors

Final approval: all authors

## Funding

VentriJect supported this study through a small project grant and supplied Seismofit® devices and consumables.

## Declarations of interest

The authors declare that they have no conflicts of interest.
